# Loss of P2Y12 Has Behavioral Effects in the Adult Mouse

**DOI:** 10.3390/ijms22041868

**Published:** 2021-02-13

**Authors:** Rebecca L. Lowery, Monique S. Mendes, Brandon T. Sanders, Allison J. Murphy, Brendan S. Whitelaw, Cassandra E. Lamantia, Ania K. Majewska

**Affiliations:** 1 Center for Visual Science, Department of Neuroscience, University of Rochester, Rochester, NY 14642, USA; Rebecca_Lowery@urmc.rochester.edu (R.L.L.); mmendes@stanford.edu (M.S.M.); brsanders@claflin.edu (B.T.S.); Allison_Murphy@urmc.rochester.edu (A.J.M.); Brendan_Whitelaw@urmc.rochester.edu (B.S.W.); Cassandra_Lamantia@urmc.rochester.edu (C.E.L.); 2 Department of Biology, Stanford University, Stanford, CA 94305, USA

**Keywords:** purines, microglia, synaptic plasticity

## Abstract

While microglia have been established as critical mediators of synaptic plasticity, the molecular signals underlying this process are still being uncovered. Increasing evidence suggests that microglia utilize these signals in a temporally and regionally heterogeneous manner. Subsequently, it is necessary to understand the conditions under which different molecular signals are employed by microglia to mediate the physiological process of synaptic remodeling in development and adulthood. While the microglial purinergic receptor P2Y12 is required for ocular dominance plasticity, an adolescent form of experience-dependent plasticity, it remains unknown whether P2Y12 functions in other forms of plasticity at different developmental time points or in different brain regions. Using a combination of ex vivo characterization and behavioral testing, we examined how the loss of P2Y12 affects developmental processes and behavioral performance in adulthood in mice. We found P2Y12 was not required for an early form of plasticity in the developing visual thalamus and did not affect microglial migration into barrels in the developing somatosensory cortex. In adult mice, however, the loss of P2Y12 resulted in alterations in recognition and social memory, as well as anxiety-like behaviors, suggesting that while P2Y12 is not a universal regulator of synaptic plasticity, the loss of P2Y12 is sufficient to cause functional defects.

## 1. Introduction

Microglia, the immune cells of the brain, have now been established as critical mediators of the physiological process of synaptic plasticity [1]. However, the identification of the molecular signals mediating interactions between microglia and other cell types or the extracellular milieu is still in its early stages. One of the emerging trends suggests there is significant overlap in the signaling mechanisms utilized by microglia across their pathological and physiological roles. Several known mediators of immune function, such as fractalkine and complement signaling [2,3] have also been implicated in the physiological role of microglia in modulating neuronal circuitry and synaptic function [4,5]. Additionally, there is increasing evidence that microglia utilize different molecular signals in a temporally and regionally heterogeneous manner, dependent on brain region [4,6,7,8,9,10] and developmental process [11,12]. Given the variety and prevalence of neurological disorders linked to alterations in neuronal circuitry and synaptic function, defining the spatiotemporal profiles of the mechanisms through which microglia shape neural circuits is important for understanding, treating, and preventing these disorders.

Signaling through purines is an evolutionarily old pathway, and within the brain different purines can be released, processed, and sensed by a multitude of different cells under both physiological and pathophysiological conditions. Increasing evidence suggests that purinergic signaling contributes to synaptic plasticity both inside and outside the CNS [13,14,15,16]. P2Y12 is a G_i/o_-coupled purinergic receptor expressed in the CNS specifically by homeostatic microglia [17]. The activation of P2Y12 via ADP has been shown to induce rapid microglial chemotaxis and the directional branching of microglial processes [18,19]. P2Y12 is highly expressed along the microglial membrane under physiological conditions [20] but is rapidly downregulated following injury [18], suggesting that P2Y12 activation allows microglia to rapidly detect changes in brain homeostasis and mount an appropriate response. While P2Y12 has largely been studied in the context of injury and disease, previous work from our lab showed that the genetic or pharmacological disruption of P2Y12 signaling abolishes changes in eye-specific cortical responses after monocular deprivation during the visual critical period while also disrupting the underlying normal interactions between microglia and synaptic elements [14]. The fact that P2Y12 is expressed at high levels in both developing and adult microglia in the absence of pathology [21] suggests that purinergic signaling through this receptor could be important for other forms of developmental and adult plasticity in different brain regions.

To examine to what extent P2Y12 is a common effector of microglial behaviors that are relevant to synaptic plasticity and circuit function, we assayed the impact of the genetic deletion of P2Y12 on early developmental processes in the lateral geniculate nucleus (LGN) and primary somatosensory cortex (S1), as well as on performance in a variety of behavioral tests relevant to disorders with synaptic pathology in adult mice. We found no alterations in *P2Y12^−/−^* mice in the developmental reorganization of retinogeniculate (RGC) projections in the LGN or the microglial infiltration and density in the barrel field of S1, suggesting that P2Y12 is not required for these developmental processes. *P2Y12^−/−^* adult mice, however, displayed a variety of alterations in learning, social interactions, and anxiety-like behavior, suggesting that the prolonged loss of P2Y12 affects the function of circuits that mediate such behaviors in adulthood.

## 2. Results

**Figure 1:**
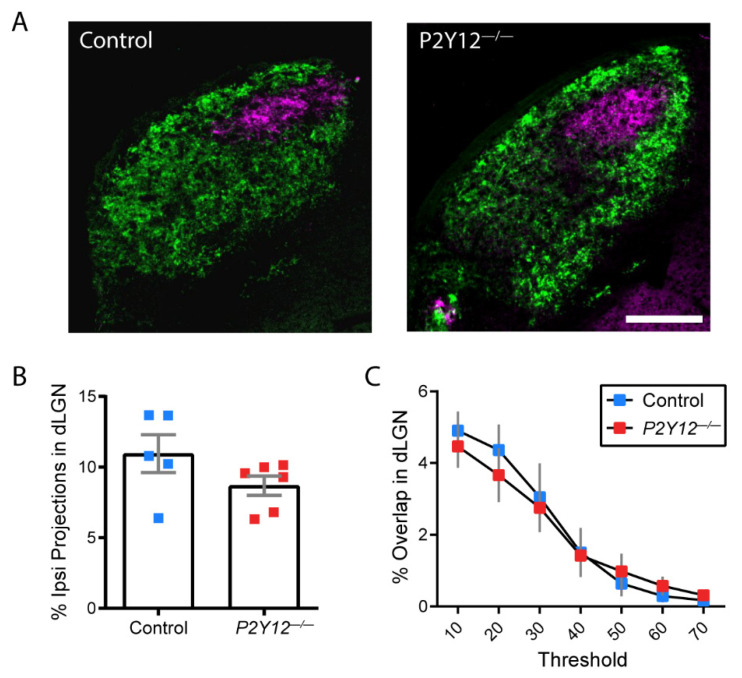
P2Y12 is not required for developmental retinogeniculate (RGC) reorganization in the lateral geniculate nucleus. (LGN): (**A**) representative images of contralateral (green) and ipsilateral (magenta) eye projections in the LGN of C57BL/6J and *P2Y12^−/−^* mice at P29. Scale bar = 500 µm; (**B**) there is no significant difference in the proportion of ipsilateral eye projections in the dorsal LGN across genotypes; (**C**) there is no significant difference in the percent of overlap between ipsilateral and contralateral eye projections in the dorsal LGN across genotypes across a range of noise thresholds. (**B**): unpaired *t*-test, (**C**): two-way ANOVA; *n* = 5–6 mice per genotype; graphs show the mean +/− SEM.

Microglia play an important role in the activity-dependent remodeling of eye-specific retinogeniculate connections in the dorsal LGN through a complement-mediated pathway that enables the internalization of RGC terminals by microglia [5]. Since P2Y12 is required for experience-dependent plasticity in the visual cortex at a later developmental stage [14], we investigated whether P2Y12 signaling also contributes to this early form of plasticity in the LGN. At P28, male and female wildtype and *P2Y12^−/−^* mice were intraocularly injected with cholera toxin B conjugated to a fluorescent tracer—Alexa597 in the left eye and Alexa647 in the right eye—to assess the projections of RGCs in each eye to the LGN. The LGN undergoes a developmental activity-dependent process whereby projections from each eye, which are initially overlapping, segregate such that they occupy eye-specific regions. We found that compared to control mice, mice lacking P2Y12 exhibit no significant differences in the area occupied by ipsilateral eye-specific projections or overlap between ipsilateral and contralateral eye-specific projections (Figure 1A–C). These findings indicate that the developmental organization of the LGN proceeds normally in the absence of P2Y12 and that contrary to its critical role in adolescent visual system plasticity, P2Y12 is not required for this early form of visual system plasticity.

**Figure 2:**
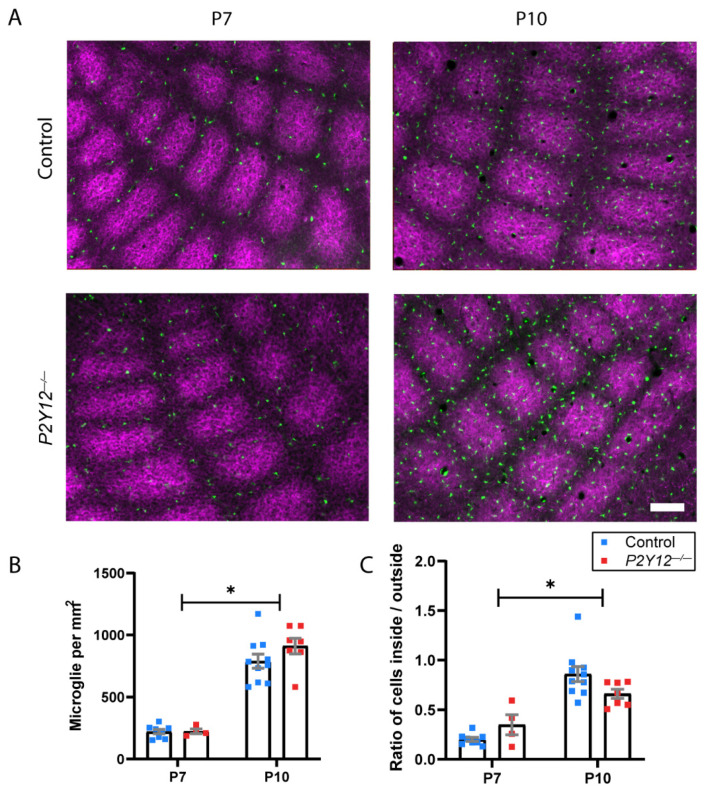
P2Y12 is not required for developmental microglial infiltration into thalamocortical axon (TCA) clusters in S1 barrel fields: (**A**) representative images of microglial (green) distribution inside 5-HTT-labeled TCA clusters (magenta) in tangential sections at P7 and P10 in *Cx3cr1^G/+^* and *P2Y12^−/−^*/ *Cx3cr1^G/+^* mice. Scale bar = 100 µm; (**B**) microglia density increases over time with no effect of genotype (*n* = 4–10 per group); (**C**) the number of microglia inside TCA clusters increases over time with no effect of genotype. (**B**,**C**): two-way ANOVA, Sidak post hoc, * *p* < 0.0001; *n* = 4–10 per group; graphs show the mean +/− SEM.

We then assayed another early developmental process which occurs in the primary somatosensory cortex (S1), whereby microglia infiltrate and distribute within the barrel structures that correspond to individual whisker inputs. This process depends on the microglial “sensome” and is delayed in the absence of Cx3cr1, another molecular mechanism involved in microglia-mediated circuit refinement, whose loss results in transient defects in synaptic maturation state [7]. We examined microglia in fixed S1 sections of *Cx3cr1^G/+^* mice and *P2Y12^−/−^*/*Cx3cr1^G/+^* mice at P7, before infiltration has begun, and at P10, when this process is complete in *Cx3cr1^G/+^* animals [7]. Microglial density was not significantly different between genotypes and significantly increased with age (Figure 2A,B; two-way ANOVA, F (1, 25) = 132.3, *p* < 0.0001). We then assayed the number of microglia within and outside the barrels, as defined by 5-HTT immunohistochemistry and compared the ratio of these numbers between genotypes. Both genotypes showed significant increases in this ratio indicative of microglial infiltration into barrels over time (Figure 2A,C; two-way ANOVA, F (1, 25) = 51.07, *p* < 0.0001). While a slight decrease in this ratio was observed in *P2Y12^−/−^* mice, this effect was not statistically significant, suggesting that P2Y12 plays a minor role in this process. 

**Figure 3:**
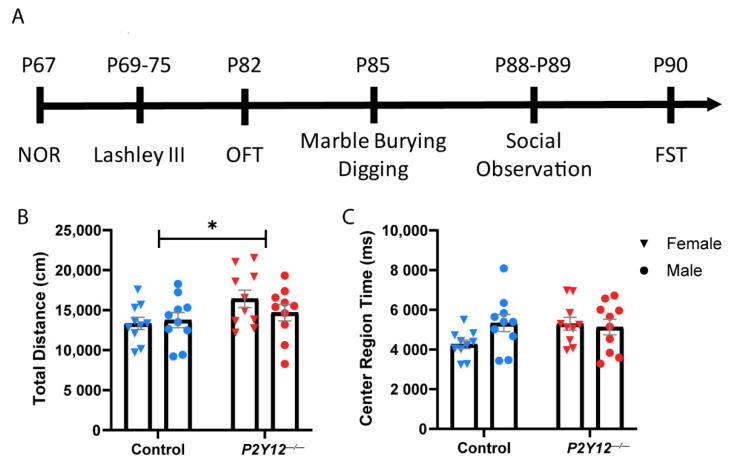
Behavioral testing of *P2Y12^−/−^* mice: (**A**) timeline of behavioral testing; (**B**) *P2Y12^−/−^* mice had a small increase in total distance traveled during the open field test; (**C**) there was no significant effect of genotype on the time spent in the center region of the open field test. (**B**,**C**): two-way ANOVA, Sidak post hoc, * *p* < 0.05; *n* = 20 mice per genotype (10 males, 10 females); graphs show the mean +/− SEM.

**Figure 4:**
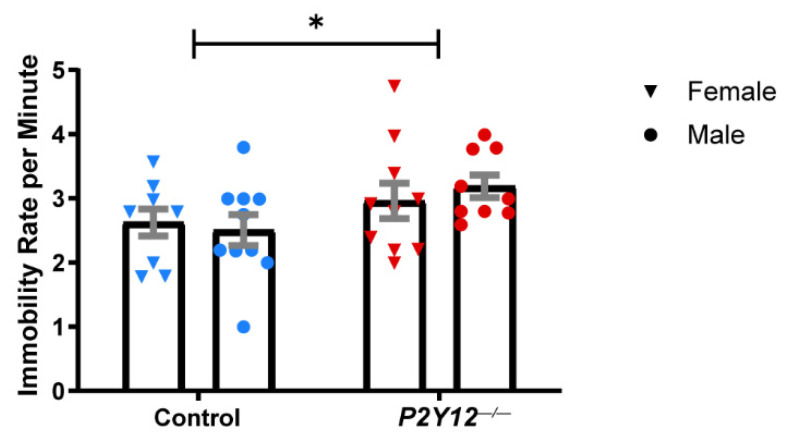
*P2Y12*^−/−^ mice exhibit higher anxiety-like behavior. *P2Y12^−/−^* mice had a higher immobility rate in the Figure 3. Two-way ANOVA, Sidak post hoc, * *p* < 0.05; *n* = 20 mice per genotype (10 males, 10 females); graph shows the mean +/− SEM.

**Figure 5:**
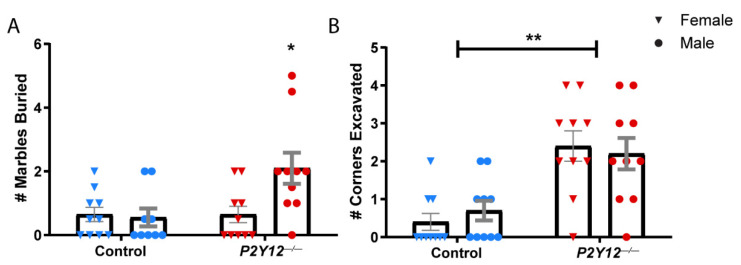
*P2Y12^−/−^* mice exhibit increased repetitive behaviors: (**A**) male *P2Y12^−/−^* mice exhibited increased burying behavior; (**B**) both male and female *P2Y12^−/−^* mice exhibited increased digging behavior. (**A**,**B**): two-way ANOVA, Sidak post hoc, * *p* < 0.05, ** *p <* 0.0001; *n* = 20 mice per genotype (10 males, 10 females); graph shows the mean +/− SEM.

Our results suggest that the loss of P2Y12 does not prevent microglia from contributing to synapse elimination in early plasticity in the LGN (Figure 1) or from being appropriately distributed within barrels during the development of the somatosensory cortex (Figure 2), despite its critical role in microglia during ocular dominance plasticity in the visual cortex [14]. This suggests that P2Y12 functions within microglia to alter microglia–synapse interactions in a heterogeneous manner, which depends on the context. To better understand how P2Y12 contributes to the maturation and function of neural circuits, we decided to assess the behavior of adult animals. We therefore subjected wildtype and *P2Y12^−/−^* mice (~P67) to a battery of behavioral tests (see Figure 3A for timeline). Because many previous studies have shown sex-specific differences in behavioral tests, we assayed male and female mice of both genotypes and all tests were run during the dark phase of the light cycle to ensure that animals were alert and ready to perform. To determine whether a constitutive loss of P2Y12 results in defects in locomotor behavior, the spontaneous activity of control and *P2Y12^−/−^* mice was assayed in the open field test. While we found a main effect of genotype with a small significant increase in total distance traveled by *P2Y12^−/−^* mice (Figure 3B; two-way ANOVA, F (1, 36) = 4.324, *p* = 0.0448), there was no significant difference in the center area exploration time (Figure 3C), suggesting a mild increase in locomotor activity with no difference in anxiety-like behavior as assayed by this simple test. To further explore anxiety-like behaviors, we performed the forced swim test (FST), whereby time to immobility was measured as an assay of behavioral despair. Both male and female *P2Y12^−/−^* mice exhibited a higher rate of freezing behavior in the FST as compared to the controls, suggesting that the loss of the P2Y12 receptor can result in increased anxiety-like behaviors (Figure 4; two-way ANOVA, F (1, 34) = 4.715, *p* = 0.0370). Repetitive behaviors, along with increased anxiety, have been described in many rodent models of neurodevelopmental disorders [22], To determine whether the absence of P2Y12 could induce repetitive behaviors, we used the marble burying test, which measures the repetition of spontaneously occurring behaviors, to assay both burying and digging behavior. Compared to the controls, male but not female *P2Y12^−/−^* mice exhibited increased burying behavior (Figure 5A; two-way ANOVA, F (1, 35) = 5.39, *p* = 0.0262; Sidak post hoc, *p* < 0.05), while both male and female *P2Y12^−/−^* mice exhibited increased digging behavior (Figure 5B; two-way ANOVA, F (1, 36) = 27.22, *p* < 0.0001). These results suggest that the absence of the P2Y12 receptor can result in increased repetitive behaviors with a sex-specific component.

**Figure 6:**
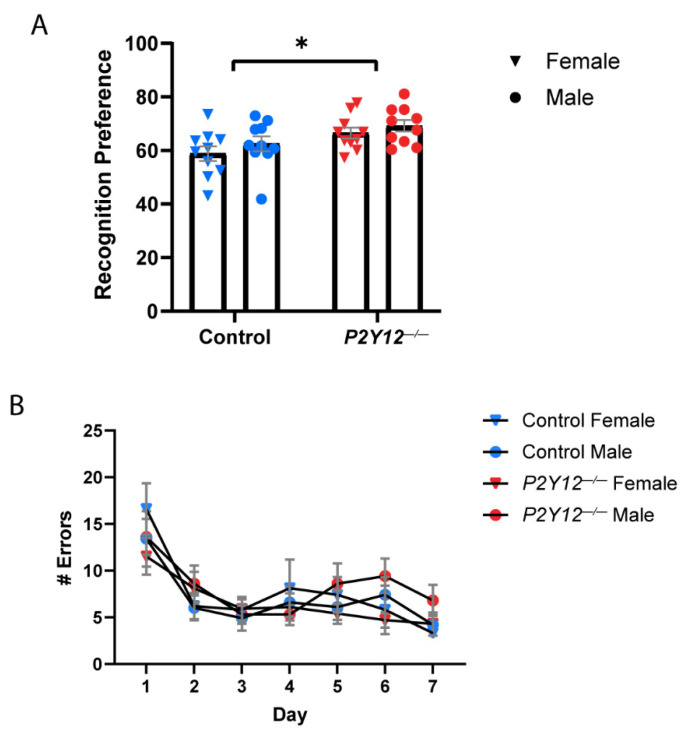
*P2Y12^−/−^* mice exhibit selective alterations in learning: (**A**) *P2Y12^−/−^* mice had a higher recognition preference for a novel object, demonstrating enhanced associative learning, but no difference in spatial learning and memory (**B**); (**A**,**B**): two-way ANOVA, Sidak post hoc * *p* < 0.01; *n* = 20 mice per genotype (10 males, 10 females); graph shows the mean +/− SEM.

To examine the impact of P2Y12 deletion on general learning and memory, control and *P2Y12^−/−^* mice underwent novel object recognition (NOR) and Lashley III maze (LIII) testing. NOR testing consisted of a 5 min habituation period the day before testing, a 10 min exploration phase to familiarize with the objects, a 2 h intersession interval, and a 10 min test phase to explore the novel object. NOR testing demonstrated a significant effect of genotype on recognition preference, with no impact of sex (Figure 6A; two-way ANOVA, F (1, 36) = 8.578, *p* = 0.0059). *P2Y12^−/−^* mice exhibited a higher recognition preference, which measures the time spent with the novel object as compared to the familiar object. This indicates enhanced novel object recognition and suggests that P2Y12 may play a role in the establishment of associative learning. The Lashley III maze was used to assay learning and memory in a low-stress environment, independent of environmental visual cues or aversive stimuli to motivate learning, as home cage destination is sufficient reward to motivate mice to learn the maze route. When comparing the number of errors made over the LIII maze test days, defined as entries into dead ends or backward travel, neither control nor *P2Y12^−/−^* mice reached the criteria for route learning. Both genotypes, however, demonstrated an early decrease in number of errors made, indicating enhanced performance. However, there was no significant effect of either genotype or sex on the number of errors made, suggesting there is no defect in spatial learning and memory in the absence of P2Y12 (Figure 6B).

**Figure 7:**
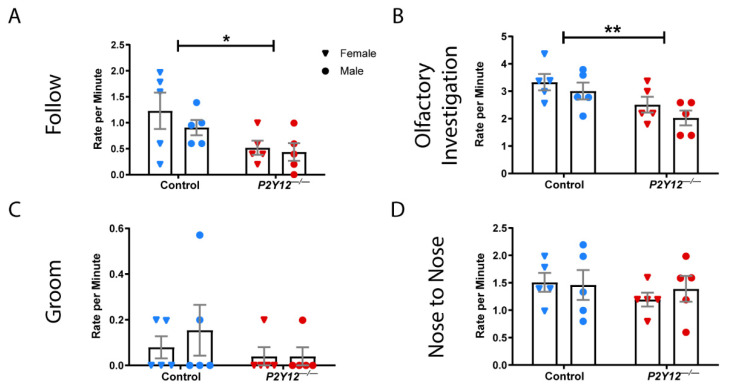
*P2Y12^−/−^* mice exhibit a subset of altered social behaviors. *P2Y12^−/−^* mice exhibited a decreased rate of following (**A**) and olfactory investigation (**B**) behaviors but not allogrooming (**C**) or nose-to-nose investigation (**D**). (**A**–**D**): two-way ANOVA, Sidak post hoc, * *p* < 0.05, ** *p* < 0.01; *n* = 20 mice per genotype (10 males, 10 females); graph shows the mean +/− SEM.

Because alterations in social interactions are characteristic of disorders with synaptic pathologies [23,24], we examined whether the absence of P2Y12 influenced social behavior. Spontaneous interactions between pair-housed mice were observed over a 10 min period following the reintroduction of the pair and scored for a set of social behaviors. Social observation demonstrated that both male and female *P2Y12^−/−^* mice exhibited a decreased rate of following (Figure 7A; two-way ANOVA, F (1, 16) = 7.281, *p* = 0.0158) and olfactory investigation (Figure 7B; two-way ANOVA, F (1, 16) = 9.571, *p* = 0.0070) over a 10 min period, but not allogrooming or nose-to-nose sniffing (Figure 7C,D). These results suggest that the loss of the P2Y12 receptor can result in a decrease in a subset of reciprocal social interactions.

## 3. Discussion

In these experiments, we showed that unlike ocular dominance plasticity in the adolescent visual cortex, P2Y12 is not required for synaptic remodeling in the early development of the LGN, a process that is known to depend on microglia, nor is it required for microglial distribution within developing barrels in the somatosensory cortex. However, in line with previous investigations [25,26], the loss of P2Y12 results in altered behavior in the adult mouse, including increased anxiety, decreased social interactions, and more heterogeneous effects on learning. This suggests that loss of P2Y12 alters neural circuits and impacts adult brain function.

### 3.1. P2Y12 as a Heterogeneous Regulator of Synaptic Plasticity

Increasing evidence suggests that microglia impact synaptic function but that the mechanisms that microglia use in their roles as synaptic architects are not stereotyped and vary by context. The contribution of signaling pathways implicated in microglial-mediated circuit refinement, such as fractalkine signaling and the complement pathway, is both time and region-dependent [4,5,7,8,9,10,11,12,14]. Similarly, we now show that P2Y12, although highly expressed by most microglia across development and across brain regions, is critical for only a subset of microglial behaviors in ways that overlap with and differ from other mechanisms. For instance, signaling through the fractalkine receptor, Cx3cr1, does not play a role in either developmental (LGN) or adolescent (ODP) forms of synaptic plasticity in the visual system [8,10], but P2Y12 is required for adolescent ODP [14] but not LGN plasticity. P2Y12 additionally differs from Cx3cr1 in that while Cx3cr1 is involved in microglial infiltration into barrel fields in S1 with subsequent transient synaptic defects [7], we found that P2Y12 was not involved in this microglial infiltration. P2Y12 also has a different pattern of function compared to C1q, since C1q is required for the developmental organization of the LGN [11] but not required for adolescent ODP [12], while P2Y12 is required for adolescent ODP [14] but not required for the developmental organization of the LGN. Given what is known about the microglial expression of these factors throughout the brain, these findings support the idea that the utilization of microglial signaling mechanisms is dependent not just on variation in microglial expression of the players in these pathways but also on the microglial state and/or signals from the environment. Overall, this study confirmed that, like other established molecular mechanisms of microglia-mediated synaptic modulation, the role of P2Y12 is heterogeneous and depends on brain region and developmental process and further exploration is needed to determine the precise spatiotemporal application of this signaling pathway.

### 3.2. P2Y12 and Behavior

Several studies have reported changes in animal behavior after the disruption of microglial pathways that contribute to synapse remodeling [27,28,29]. Recently, a pair of studies examined the role of P2Y12 in fear acquisition and anxiety-like behaviors, finding that P2Y12 is required for innate fear learning and its loss increases anxiety-like behaviors [25,26]. In line with these results, we found enhanced anxiety-like behaviors in *P2Y12^−/−^* mice as measured by FST, underscoring the function of P2Y12 in mediating anxiety responses. It is important to note that both our study and that of Peng et al. found no increased anxiety-like behavior in the open field test, although Zheng et al. found increased anxiety-like behavior using this test. Additionally, while Peng et al. found no change in locomotor behavior and Zheng et al. found decreased locomotor behavior, we found slightly increased locomotor behavior. These differing results likely occurred as a result of differences in experimental parameters, pre-testing environment habituation, the sex of animals tested, and the use of different genetic strains which included global vs. conditional knockout strategies. The use of the congenital knockout mouse line [30] in our study and that of Zheng et al. introduces the possibility that compensatory or developmental effects may impact our results, convolving the effects of P2Y12 on circuit development with those on the function of adult circuits. As such, it would be interesting to repeat these experiments in the new, inducible *P2Y12^−/−^* mouse line [25]. However, both constitutive and inducible knockout mice showed enhanced innate fear responses suggesting that P2Y12 may impact how microglia and neurons interact in the adult to shape behavior [25]. The conditional studies also suggest that peripheral P2Y12 loss does not dominate the effects on behavior and that these are indeed mediated by microglial P2Y12. Additionally, the deletion of P2Y12 may result in compensatory changes in P2Y13 as it is similarly highly expressed in microglia and may have similar functions. However, the inverse deletion of P2Y13 does not result in altered P2Y12 expression nor compensatory functioning in the brain [31], suggesting that these two similar receptors are independently regulated.

In addition to the previously demonstrated role of P2Y12 in fear and anxiety-like behaviors, we demonstrated certain alterations in recognition memory, social interactions, and repetitive behaviors. While we found no impact on general spatial memory, we found enhanced recognition of a novel object. While this could indicate improved associative learning in the absence of P2Y12, it could also potentially indicate a difference in the ability of *P2Y12^−/−^* mice to habituate to a new environment. A study examining the role of the hippocampus in NOR memory found that the reversible inactivation of the hippocampus resulted in enhanced recognition memory, similar to our findings in *P2Y12^−/−^* mice [32]. However, in this study, enhanced recognition memory was caused by a defect in habituation to a novel context, as increasing the habituation period prior to training restored recognition memory to control levels. Given that *P2Y12^−/−^* mice have demonstrated defects in hippocampal signaling [25] and the hippocampus is a critical component of functional habituation [33], a similar defect in habituation could underlie our NOR results. Notably, an increase in the time needed to achieve habitation to a novel stimulus is a phenotype seen in ASD [34,35,36], along with decreased social interactions and increased repetitive behaviors, which are defining characteristics of ASD and other synaptopathologies [23,37]. The coexistence of these behavioral features in *P2Y12^−/−^* mice suggests that P2Y12 deletion may alter synaptic function to mimic some aspects of neurodevelopmental disorders. In addition, these human disorders are characterized by sex-specific incidence and symptoms. However, we found a minimal impact of sex in our study, with only one test showing increased marble-burying behavior in *P2Y12^−/−^* male but not female mice. Overall, our findings agree with previous studies of *P2Y12^−/−^* mice, suggesting that purinergic signaling in microglia mediates microglia–neuron interactions in the adult brain shaping behavioral responses. Taken with our results showing that P2Y12 does not play a large role in two developmental microglial functions in the visual thalamus and somatosensory cortex, our behavioral studies suggest that P2Y12 is used by microglia in specific contexts, brain regions, and developmental time periods. 

### 3.3. Purinergic Signaling and Synaptic Plasticity

P2Y12 is highly expressed in the membrane of homeostatic microglia throughout development and adulthood [20], where it serves to detect elevations in extracellular ADP which occur as a result of cellular injury rapidly recruiting microglia to areas of damage [18]. How P2Y12 contributes to microglial interactions with synapses in the absence of injury, however, is still not known. It has been demonstrated that P2Y12 mediates directed process motility [38] and microglial process interactions with synaptic elements [14], suggesting that the loss of P2Y12 would interfere with the ability of microglia to receive activity-dependent signals to translocate to, and potentially modify, a target synapse. Several studies suggest that NMDA activity at synapses recruits microglia through P2Y12 [39,40]. However, the source of the purines is hard to determine as purines are rapidly hydrolyzed and can be released by many cell types, including neurons, astrocytes, and microglia [41,42,43,44,45]. ATP released by neurons could directly signal activity levels to microglia, using an ATP concentration gradient to attract microglial processes to synaptic elements [46], and there is evidence for ATP co-release in synaptic vesicles [45,47]. Alternatively, astrocytes could serve as an intermediary, reading out synaptic activity [48,49,50,51,52] and releasing purines to signal to microglia. Indeed, microglial directed process motility is influenced by ATP from astrocytes [46]. Additionally, microglia release ATP via exocytosis [44] and this self-released ATP can contribute to the directed long-range migration of microglia via autocrine signaling through P2Y receptors [53]. Thus, microglia could use autocrine ATP signaling to self-propagate the motility of their processes via binding to P2Y12 while other signals indicate specific synaptic targets. Depending on the method of intercellular signaling, purines could serve several functions in the process of synaptic modulation, including allowing microglia to sense neuronal activity [39,40], recruiting microglial processes to synaptic elements either alone [14,18,39] or in combination with the go and stop signals that allow synaptic specificity [5,54], mediating the pruning of synapses by microglia [5,14], or recruiting microglial pathways that in turn alter neuronal activity [55,56]. An important aspect in determining cell–cell interactions and the precise function of purinergic signaling in the process of synaptic modulation will be to identify the source of the purines that activate the P2Y12 receptor on microglia.

Changes in neuronal function and neurotransmission have recently been described in *P2Y12^−/−^* mice, both of which could have profound effects on behavioral outcomes. *P2Y12^−/−^* mice were found to have both enhanced neuronal excitability [25] and decreased noradrenergic signaling [26]. As disruptions in noradrenergic signaling negatively impact microglial surveillance and associated synaptic plasticity [57,58], both of these alterations could lead to defective neuronal circuitry and subsequent behavioral deficits. Additional research on the interplay between purinergic and adrenergic signaling, which have been proposed to have antagonistic effects on microglial process motility [59], as they relate to alterations in neuronal circuitry could further reveal the precise mechanisms underlying the observed behavioral defects in *P2Y12^−/−^* mice.

Overall, our findings suggest that P2Y12 is not a critical player in all microglial functions that involve microglia–neuron communication, despite its high expression throughout development and adulthood. While not a universal regulator of plasticity, P2Y12 likely plays a role outside of the visual system in brain regions underlying associative learning or habitation, social interactions, and anxiety-like behaviors. Future studies should investigate the potential molecular and cellular deficiencies in these regions resulting from P2Y12 deletion. It is important to thoroughly understand the role of P2Y12 as it pertains to regulating circuit development and synaptic plasticity given the proposed potential of targeting purinergic signaling for therapeutics in cognitive dysfunction and the existing prevalence of the use of P2Y12 receptor antagonist medication as an anti-platelet drug.

## 4. Material and Methods

### 4.1. Experimental Subjects

Experimental protocols were carried out in strict accordance with the University of Rochester Committee on Animal Resources (UCAR) and conformed to the National Institute of Health’s “Guide for the Care and Use of Laboratory Animals, 8th Edition, 2011.” The examination of retinogeniculate projections in the lateral geniculate nucleus (Figure 1) was carried out between postnatal days (P)28 and P29, as the reorganization of these projections is complete by this time and the final organization of eye-specific layers can be assessed. Experiments examining microglial infiltration into thalamocortical axon (TCA) clusters (Figure 2) were carried out at P7 and P10 to replicate previously published methods [7]. Behavioral experiments began when mice were P67 and ended by P90 (Figure 3, Figure 4, Figure 5, Figure 6 and Figure 7). Both female and male mice were included in all experiments and all mouse lines were generated on a C57BL/6J background. C57BL/6J (Jackson Labs), P2Y12^−/−^ ([30]; kindly provided by M. Nedergaard, URMC, Rochester, NY, USA), and Cx3cr1-EGP [60] mouse lines were used. All animals were bred in-house under a standard 12/12 h light/dark cycle and fed ad libitum with standard chow. For behavioral experiments, the animals were housed in a reverse light cycle room and all behavioral assessments were carried out during the dark phase. Experiments and analyses were performed masked to genotype.

### 4.2. Behavioral Testing

Behavioral testing was performed from when mice were P67 and concluded before P90 to prevent introducing the confound of age. Behavioral tests were performed in the following order: novel object recognition, Lashley III maze, open field test (OFT), marble burying/digging, social observation, and forced swim test. The order of testing was designed to minimize the influence of confounding factors, with the forced swim test performed last to prevent the effects of stress from influencing the results of the other behavioral tests. The order and timing of the testing was the same for all subjects, such that mice of both genotypes were tested together, at the same time and at the same age. However, we cannot be certain that the order of testing, as well as any developmental effects of the three-week period during which testing was administered, did not affect the results reported.

Open field activity: To test ambulatory activity, a single experimental mouse was placed inside a Plexiglas test environment (17.0 in × 17.0 in), where three 16-beam I/R arrays measured the position of the animal at 50 ms intervals over the course of 60 min. At the end of the session, the mouse was placed back in its home cage and chambers were cleaned between sessions. 

Novel object recognition: The NOR test uses the mouse’s preference for novel objects to examine recognition memory. A single experimental mouse was placed in an open box (35 cm long × 35 cm wide × 35 cm high) in a dimly lit room for 5 min to habituate to the box and then immediately returned to its home cage. Twenty-four hours later, 2 identical objects were fixed in place in adjacent corners of the box. The mouse was then re-exposed to the box and allowed to explore the novel objects for 10 min and then returned to its home cage. Two hours later, one object was replaced by a different (novel) object. The mouse was then re-exposed to the box for 10 min and time spent interacting with the 2 objects was measured. All experiments were videotaped for scoring purposes. The box and objects were cleaned in between animals.

Lashley III maze: The Lashley III Maze is used to assess learning and memory without the use of visual cues, food/water deprivation, or aversive stimuli. Mice were allowed 10 min to navigate a maze consisting of four chambers divided into segments in order to reach their home cage. If they failed to complete the maze in the allotted 10 min, they were removed and returned to their home cage. Trials were run once a day over a 7-day period. All trials were recorded for the scoring of errors (entry into dead-end segments or re-entry into previously traveled segments) and the maze was cleaned between mice to remove any olfactory cues.

Social observation: This procedure measures spontaneous social interaction between the focal subject and a stimulus animal. The focal mouse was placed in a Plexiglas test environment (12.0 in by 12.0 in) with another stimulus mouse. Subject interactions over a 10 min period were recorded and videos were later scored by an experience masked observer from a prewritten ethogram of social behaviors. The arena was cleaned between each subject.

Forced swim test: Forced swim testing was used to measure anxiety-like behavior. Mice were placed in a 5 L beaker of room temperature water for a maximum period of 5 min without the opportunity to escape. Testing was video recorded and scored for immobility events by an experienced masked observer. The beaker was cleaned between each subject.

Marble burying and digging behavior: A 28 cm × 40 cm plastic box was filled with 4 inches of bedding. Twenty marbles were positioned in a 5 × 4 grid. An individual experimental mouse was gently placed in the corner of the box and gently removed from the box after 20 min, taking care not to disturb or artificially cover marbles. Three masked observers scored the number of marbles at least two-thirds buried in bedding and the number of corners excavated more than 2 inches. An average score per animal was generated based on the three observers.

### 4.3. Intraocular Injections

P28 C57BL/6J and *P2Y12^−/−^* mice were anesthetized with a mixture of fentanyl (0.05 mg/kg, i.p.), midazolam (5.0 mg/kg, i.p.) and dexmedetomidine (0.5 mg/kg, i.p.). A 33-gauge Hamilton syringe was used to inject anterograde tracer (CtB-AlexaFluor594 or CtB-AlexaFluor647, 0.5% solution in sterile saline, Thermo-Fisher, Cat# C34777, Cat# C34778) into the vitreous fluid. Twenty-four hours later, at p29, following the injection with Euthasol (Virbac), the mice were transcardially perfused with 0.1 M phosphate-buffered saline (PBS) followed by 4% paraformaldehyde (PFA) in 0.1M PBS. Brains were harvested and post-fixed in 4% PFA overnight followed by cryo-protection with 30% sucrose in 0.2 M phosphate buffer (PB).

### 4.4. LGN Projection Analysis

Coronal brain sections were cut on a freezing microtome. For each animal, 2 sections were analyzed from the middle third of the dorsal lateral geniculate nucleus (dLGN) of each hemisphere [61,62]. Sections were imaged using a UPlanApo 4x/0.16NA objective on a BX51 Olympus scope (Olympus, Tokyo, Japan) mounted with a Spot Pursuit RT color digital camera (Diagnostic Instruments, Sterling Heights, MI, USA) at uniform exposure settings. Thresholded images were analyzed offline in ImageJ. The area occupied by ipsilateral projections was quantified as the number of pixels present in the fluorescence channel corresponding to the dye injected into the ipsilateral eye normalized by the number of pixels in the entire dLGN. The percent overlap was quantified as the number of pixels overlapping in both channels, measured over a range of noise thresholds [63].

### 4.5. Immunohistochemistry

Following injection with Euthasol (Virbac, France), mice were perfused transcardially with 0.1 M phosphate buffered saline (PBS) followed by 4% paraformaldehyde (PFA) in 0.1 M PBS. Following overnight fixation in 4% PFA at 4 °C, brains were cryoprotected with 30% sucrose in 0.2 M phosphate buffer (PB). Tangential sections (for 5-HTT reactivity) were cut on a freezing microtome (Microme; Global Medical Instrumentation, Ramsey, MN, USA) at 50 μm thickness into cryoprotectant. Sections were processed free-floating at room temperature (RT). Briefly, the sections were rinsed in 0.1 M PBS, incubated for 20 min in a 3% hydrogen peroxidase solution and for 1 h in blocking buffer. Sections were then incubated in a primary antibody solution (anti-5-HTT, 1:1000, Calbiochem, Cat# PC177L) for 24 h at 4 °C. Following primary antibody incubation, the sections were rinsed with 0.1 M PBS and incubated for 4 h at RT in a secondary antibody solution (Alexa-Fluor 594, 1:500, Invitrogen, Cat# A-21207). Following a final rinse in 0.1 M PBS, sections were mounted on slides and coverslipped with Prolong Gold Antifade Reagent (Molecular Probes, Carlsbad, CA, USA, Cat# P36934).

### 4.6. Barrel Field Analysis

For the analysis of microglial infiltration into TCA clusters, tangential sections through layer IV of S1 were imaged using a 10x, 0.30 NA objective on a BX51 Olympus scope (Olympus, Tokyo, Japan) mounted with a Spot Pursuit RT color digital camera (Diagnostic Instruments, Sterling Heights, MI, USA) at uniform exposure settings. An intrinsic GFP signal was used to visualize microglia, as in [7]. Image analysis was performed offline in ImageJ, where the number of microglial cells inside the TCA cluster (defined by that TCA cluster’s borders visualized with 5-HTT immunoreactivity) and outside the TCA clusters (defined by the borders of all neighboring TCA clusters) were quantified to determine the ratio of microglia inside to outside the TCA clusters. Analysis was performed in 5–6 barrels per animal and averaged.

### 4.7. Statistical Analysis

Statistics were carried out in Graphpad Prism 8. Comparisons were performed using unpaired t-tests or two-way ANOVA with Sidak post hoc tests for multiple comparisons as appropriate. Grubb’s Outlier test was used to identify outliers and one outlier was removed from the LIII data. All data points represent individual animal averages and are presented as the mean +/− SEM.

## Data Availability

The data presented in this study are available on request from the corresponding author.
